# The Evolution of Mutualism in Gut Microbiota Via Host Epithelial Selection

**DOI:** 10.1371/journal.pbio.1001424

**Published:** 2012-11-20

**Authors:** Jonas Schluter, Kevin R. Foster

**Affiliations:** 1 Department of Zoology, University of Oxford, Oxford, United Kingdom; 2 Oxford Centre for Integrative Systems Biology, University of Oxford, Oxford, United Kingdom; Cornell University, United States of America

## Abstract

The human gut harbours a large and genetically diverse population of symbiotic microbes that both feed and protect the host. Evolutionary theory, however, predicts that such genetic diversity can destabilise mutualistic partnerships. How then can the mutualism of the human microbiota be explained? Here we develop an individual-based model of host-associated microbial communities. We first demonstrate the fundamental problem faced by a host: The presence of a genetically diverse microbiota leads to the dominance of the fastest growing microbes instead of the microbes that are most beneficial to the host. We next investigate the potential for host secretions to influence the microbiota. This reveals that the epithelium–microbiota interface acts as a selectivity amplifier: Modest amounts of moderately selective epithelial secretions cause a complete shift in the strains growing at the epithelial surface. This occurs because of the physical structure of the epithelium–microbiota interface: Epithelial secretions have effects that permeate upwards through the whole microbial community, while lumen compounds preferentially affect cells that are soon to slough off. Finally, our model predicts that while antimicrobial secretion can promote host epithelial selection, epithelial nutrient secretion will often be key to host selection. Our findings are consistent with a growing number of empirical papers that indicate an influence of host factors upon microbiota, including growth-promoting glycoconjugates. We argue that host selection is likely to be a key mechanism in the stabilisation of the mutualism between a host and its microbiota.

## Abstract 2

A computer model of the gut shows how a host can readily select friendly bacteria over harmful bacteria through a process called “selectivity amplification.”

## Author Summary

The cells of our bodies are greatly outnumbered by the bacteria that live on us and, in particular, in our gut. It is now clear that many gut bacteria are highly beneficial, protecting us from pathogens and helping us with digestion. But what prevents beneficial bacteria from going bad? Why don't bacteria evolve to shirk on the help that they provide and simply use us as a food source? Here we explore this problem using a computer model that reduces the problem to its key elements. We first illustrate the basic problem faced by a host: Whenever beneficial bacteria grow slowly, the host will lose them to fast-growing species that provide no benefit. We then propose a solution to the host's problem: The host can use secretions—nutrients and toxins—to control the bacteria that grow on the epithelial cell layer of the gut. In particular, our model predicts that the epithelial surface acts as a “selectivity amplifier”. The host can thereby maintain beneficial bacteria with only small amounts of weakly selective secretions. Our model fits with a growing body of experimental data showing that hosts have diverse and important influences on their gut bacteria.

## Introduction

Many microbial species live on or are associated with epithelia of multicellular organisms. Examples range from plants and soil bacteria interactions in the rhizosphere where plant secretions affect the composition of bacterial communities [1],[2], through the light organs of marine animals in which specialised symbiotic bacteria are cultivated by the host [3]–[5] to many surfaces of the mammalian body [6].

Every human is home to roughly 100 trillion bacterial cells, collectively called the microbiota. The majority of these cells reside in the human gastrointestinal tract and, in particular, in the large intestine [7]. Here, bacteria can have beneficial effects such as the digestion of complex carbohydrates, colonisation resistance against invading pathogens, maturation of the adaptive mucosal immune system and immune cells, and the production of secondary metabolites, including vitamins [8]–[10]. However, these activities are not performed by all species, and the species composition of the microbiota in a healthy human is clearly distinct from bacterial communities in other environments [11]. Moreover, various diseases correlate with disturbances in the species composition of the microbiota [6],[10]. It is clear then that the gut community has the ability to both help and harm the host. Despite the potential for harmful effects of the gut microbiota, the major class of interaction with the host appears to be one of mutualism, whereby both sides benefit from the interaction. The evidence for host benefits comes both from our understanding of the metabolic services that the gut microbiota provides and studies of germ-free animal models [6],[12]–[18].

There is a growing literature on the evolution of mutualisms among species, both theoretical and empirical, which emphasizes a number of key factors required for the evolutionary stability of mutualisms [19]–[22]. Most relevant for the gut microbiota is the issue of having multiple genetically different individuals on one side of the mutualism (microbes) involved in a single interaction with the other (host) [19],[20],[22]. On the side with multiple genotypes, this can lead to the loss of helpful mutualistic genotypes, whenever non-helpful genotypes are more competitive. How is such potential conflict among partner species resolved in other systems? Theory predicts a central role for partner choice: the selection of the best mutualistic partners by a focal species [22]. Moreover, partner choice is widespread in nature with evidence from many different systems [21],[22] including leaf cutter ants and their fungus [23], legumes and rhizobia [24], and the mutualism between the bobtail squid and the luminescent bacterium *Vibrio fischeri* [25]. The predominance of partner choice mechanisms in other systems begs the question: What is the role of partner choice in the mammalian gut?

The sheer diversity of microbial species in the mammalian gut shows that hosts do not select for one or two partner species, as occurs in some mutualisms. In addition, there is a clear environmental effect on microbial species composition in the form of host nutrient intake [26],[27]. Nevertheless, there are also a range of mechanisms by which vertebrate hosts affect their microbes more directly. In particular, the intestinal epithelium produces a wide range of secretions that help to maintain the barrier between the gut lumen and host tissues [9],[28]–[31]. Central to this barrier is mucus secretion [32]–[35] that limits the direct access of bacteria to the epithelium [36]. The mucus becomes less dense, however, as it moves upwards away from the epithelium and bacteria grow in the upper layers that can feed on carbohydrates such as fucose, which the host adds to the mucus proteins [37]–[40].

The host also secretes a range of antimicrobials into the mucus, including defensins. Mucosal community composition has been studied in mice that lack an enzyme required for murine alpha-defensins but secrete human alpha-defensin [31]. The observed changes in community composition, in combination with other studies, led to the conclusion that defensins are essential regulators of intestinal microbial ecology (for a review, see [41]). More work is now required to understand the exact role of defensins as a selective agent of the microbiota. In particular, the defensins of the small intestine have been the primary focus of research, and the effect of defensins in the large intestine is less well understood. Moreover, studies have shown that production and activation of defensins can themselves be dependent on the resident microbiota [42],[43], which opens the way for feedback loops between the host and its microbiota. In addition to defensins, the adaptive immune system also has the potential for selective effects. B-cell-derived immunoglobulin A (IgA) is considered the most likely host secretion to affect the localization, growth, and composition of the microbiota [29],[44],[45].

While it is clear that epithelial secretions can affect the microbiota, the primary role is often assumed to be as a simple barrier between the lumen and host tissues [46],[47]. However, there is evidence that epithelial secretions differentially affect different strains and species. Sugars like fucose are more easily utilized by some microbial species than others [37],[38],[40], and defensins and IgA have biased effects on the microbiota [14],[29],[31],[45]. Such findings suggest that host secretions might help to control the composition of the resident microbiota [41],[48]. Indeed, it has even been suggested that control over a wide array of non-pathogenic microbes is the primary reason why adaptive immunity first evolved [49]. Despite this, we understand very little about how the host might in practice select for particular microbial strains or species.

Here we build a model to evaluate the potential of a host to select their microbiota. Ecologies like the mammalian gut are extremely complex dynamical systems and will require a central role for theoretical approaches if we are to dissect their complexity [50],[51]. We have, therefore, developed a new model of host-associated microbial communities with the goal of bringing an evolutionary perspective to the study of host–microbiota interaction. Our model is relatively complex in that it includes realistic features such as mechanistic interactions among cells, spatial structure, and chemical gradients. However, it greatly simplifies the full complexity of the gut and is not intended as a complete description. We hope to show, nevertheless, that one can gain new understanding by the application of such simplifications to the problem of the host–microbiota interaction. In particular, our study reveals three key findings. First, we demonstrate the problem of multiple genotypes on one side of a mutualistic partnership, which renders the host–microbiota mutualism intrinsically fragile. Second, we show that a solution to this fragility is host selection: The epithelium–microbiota interface acts as a selectivity amplifier that can quickly shift the composition of the microbiota at the interface. Finally, we show that central to the selectivity is the provision of nutrients, and not just antimicrobial factors, by the host. Our results suggest a host's epithelium is a remarkable environment for partner choice, which is well suited to control bacterial community composition.

## Results

**Figure 1:**
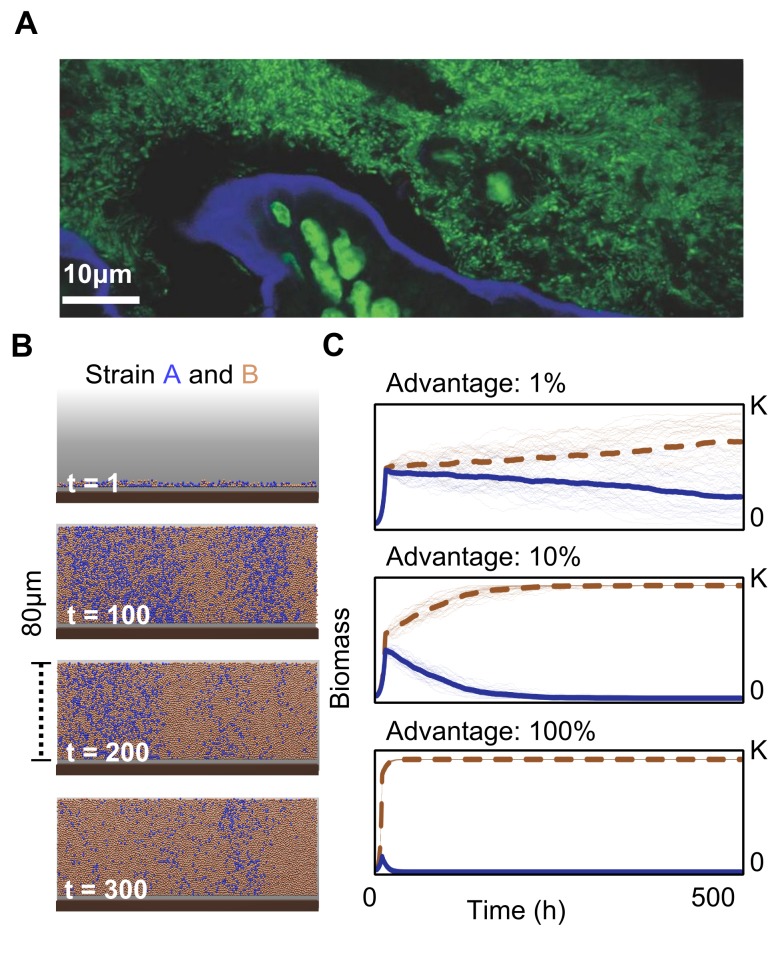
Microscopic image and simulations of microbial growth near a host epithelium. (A) Confocal fluorescence image of bacteria growing in the lumen on top of host epithelial cells. Sample taken from the cecum of a laboratory mouse, where there has been no intentional manipulation of the animal's microbiotia. Epithelial and bacterial cells in green (DNA stained with Sytox green), and the epithelial border brush in blue (actin stained with Alexa-647-phalloidin) from [83]. (B) Simulation of bacterial growth on host epithelium; brown bacterial cells (strain *B*) have a 1% growth rate advantage over blue bacterial cells (strain *A*). Even with a modest growth rate advantage, strain *B* succeeds as strain *A* is slowly washed out. (C) Thirty independent simulations of bacterial competition. Development of biomass of strain *B* (brown dashed) and *A* (blue) with growth rate advantages for strain *B* of 1%, 10%, and 100% and environmental capacity K. The thick lines are mean values.

We model a bacterial community containing two strains, which is growing on the host epithelium (Figure 1) where cells are represented by spheres that consume nutrients, grow, and divide (Materials and Methods, Table S1, Figure S1).

### Host–Microbiota Mutualism Is Fragile

Our first goal is to evaluate the potential effects of differences in growth rates between strains under the simplest of conditions, and then build in increasing complexity in order to understand the key factors at play. We denote two bacterial competitors *A* and *B*, where *B* divides more rapidly than *A* (Figure 1B). These two strains can either represent two members of one species that differ only in their interaction with the host or two different species that differ in other ways. As such, the model can be viewed from either an evolutionary (genotypes within a species) or ecological (species within a community) perspective. We return to the differences between these two scenarios in the Discussion. While we only model two strains, the model also approximates more diverse communities in which there is selection for a set of beneficial ecotypes where each “strain” would then represent multiple strains with similar phenotypes.

These simple models show the potential power of competition in a host-associated microbial community. Figure 1C shows the increase in frequency of the fittest species over time in the epithelial community. Here and in the majority of subsequent figures, we show time as an axis. One reason we do this is because it is impractical to run all simulations until the final frequencies of the two strains have been established, especially for very small differences in fitness. Nevertheless, we expect in the majority of cases that one strain will ultimately dominate the system (see following section and Text S1 for exceptions). Indeed, even for a modest difference in the growth rate among strains (e.g., 10%), a faster growing strain rapidly reduces the slower growing strain to negligible frequency in tens of generations (Figure 1C). This corresponds to a few days for species like *E. coli* in a mammalian gut [52]. The constant removal of cells leads to thinning out and eventual eradication of the slower growing strain *A* near the epithelium (Figure 1B,C). For larger difference in growth rate, such as *B* doubling at twice the rate of *A*, the eradication of *A* occurs in a few generations.

**Figure 2:**
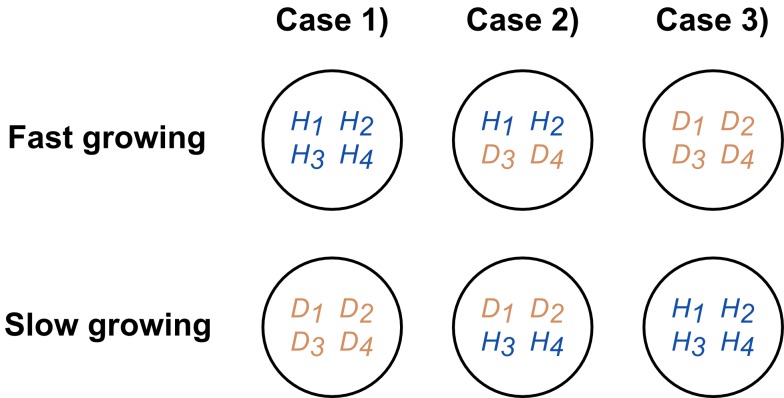
Cartoon to illustrate the potential problem faced by a host. Three scenarios are shown for four helpful strains (*H*) and four detrimental strains (*D*) that occupy four different niches, 1 to 4. Two extreme cases exist: beneficial strains grow faster in all niches (case 1) or all detrimental stains grow faster in all niches (case 3). In the first case, no partner choice is required, as natural selection favours the beneficial strain throughout all niches. However, any deviation (case 2 or 3) from this means that the host will experience a sub-optimal microbiota.

This demonstrates the fundamental problem faced by a host when having multiple possible genotypes competing for a niche where a mutualistic species could exist. Whenever the most beneficial bacteria do not grow the fastest, competition between bacterial genotypes will lead to the loss of mutualistic strains within the host and thus a suboptimal microbiota composition (Figure 2). But is it possible that mutualistic species are, without exception, intrinsically faster growing than non-mutualist species? If anything, the reverse is expected. Recent phylogenetic work shows that species from healthy guts tend to cluster with species from complex and relatively slow-growing communities [53]. By contrast, bacteria of infants and unhealthy guts tend to cluster with bacteria from fast-growing pioneer communities. In an entirely neutral host that does not exert any control over the bacterial composition, therefore, our model predicts that the mutualism between bacteria and a host is intrinsically fragile.

### Epithelial Selection Dominates Lumen Selection

So far, there is little spatial structure in our model, and we confirmed that our first results correspond to a well-mixed (no spatial resolution) ordinary differential equation model of evolutionary competition (Text S1, Figure S2). We next extend the simulations to introduce more realism and calculate nutrient levels as a function of space and time. As cells divide, they use up nutrients such that nutrient concentration is depressed as one moves away from the nutrient source and into a group of dividing cells. These solute gradients are known to be important in natural bacterial groups and can have strong influences on community structure and composition [54]–[56]. In our case, there is the potential for two solute gradients, one from the lumen direction and one from the host epithelium direction. Our question is then: How do selective compounds from the epithelium and from the lumen influence the composition of this bacterial community?

**Figure 3:**
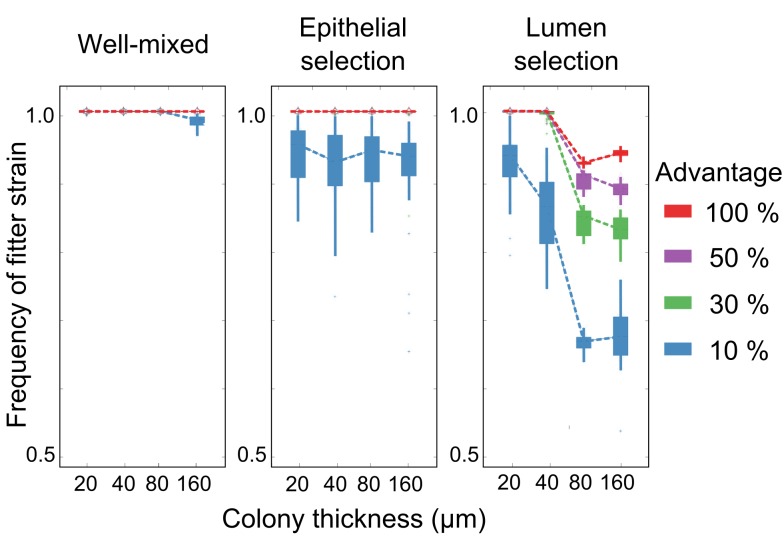
Epithelial nutrients have more effect on a bacterial community than lumen nutrients. Box plots show the final frequency of a faster growing strain after 12 d as a function of microbial community thickness, where the growth rate advantages of the fitter strain range from 10% to 100%. Well-mixed: No gradients of nutrients exist (Figure 1B,C). Epithelial selection: Nutrients exclusively diffuse into the colony from the host epithelium. Lumen selection: Nutrients exclusively diffuse into the colony from the lumen. Dashed lines connect mean values of 30 independent simulations. The total nutrient influx into the system from the host or the lumen is kept identical. Results agree with a steady-state solution of a simplified ODE model (Figure S2).

Compared to the well-mixed case, the ability of nutrients to select for one strain over the other is reduced in the presence of solute gradients because not all cells have access to nutrients. With less reproduction, natural selection is less powerful. However, more striking is that lumen nutrients exert a much weaker selective effect than epithelial nutrients. This suggests a bias that may empower the host to affect the microbial communities growing on the epithelial surface (Figure 3). What causes this difference? When the epithelium secretes nutrients, growth occurs at the base of the bacterial colony, which can affect the whole bacterial community. By contrast, lumen selection from the opposite direction preferentially affects cells that are about to be sloughed off, which limits the effect of lumen nutrients on cells at the base of the bacterial community.

The inhibition of lumen selection only occurs beyond a certain thickness of the bacterial community (Text S1, Figure S2). While it is difficult to measure the thickness of these bacterial communities in vivo, the range of thicknesses used in our model are consistent with the outer mucus layer of mice and rats [32]. A corollary of these results is that selection from the lumen should be weakened by growth near the epithelium. Hence, we further show that the addition of non-selective nutrients at the epithelium strongly inhibits lumen selection (Figure S3B). By contrast, additional non-selective lumen nutrients do not affect the ability of epithelial nutrients to select for one strain over the other.

**Figure 4:**
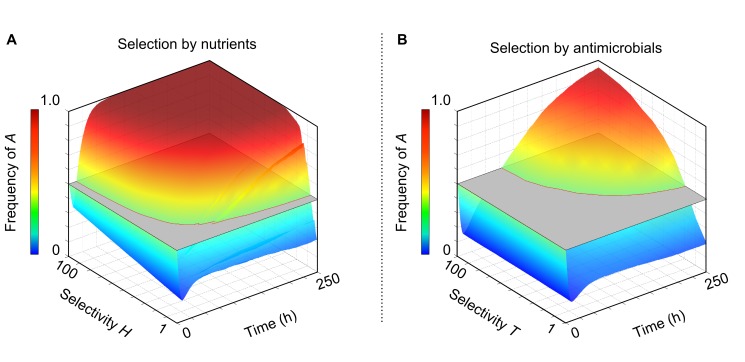
Selectivity amplification by the host epithelium. Weak epithelial selection dominates strong lumen selection. Strain *B* has a 100% growth rate advantage on nutrients from the lumen, and lumen nutrients are five times the concentration as epithelial nutrients. Grey planes mark the starting frequency of the two strains (0.5). (A) Host nutrients provide growth rate advantages to strain *A* ranging from 1% to 100%. (B) The host secretes antimicrobials that preferentially kill strain *B*; susceptibility advantages for strain *A* range from 1% to 100%. Host-secreted nutrients are also provided that neutral. In (A) and (B) strain *A* outcompetes strain *B* for all but the smallest selective advantages.

Our model predicts that the physical layout of the gut epithelium environment allows host secretions to have disproportionately strong effects. We next test this by pitting the two sources of nutrients against one another. We assume that epithelial nutrients select for strain *A*, whereas lumen nutrients select for strain *B*, simulating a scenario in which the slow growing strain *A* would be lost without host selection. We present a conservative case in which epithelial nutrients are both less abundant and less selective. Specifically, lumen nutrient concentrations are five times higher than epithelial nutrients and the growth rate advantage of strain *B* on lumen nutrients (100%) is always higher than or equal to the (varied) growth rate advantage of strain *A* on epithelial nutrients (Figure 4A).

Initially, strain *B* outgrows strain *A* as the former's overall growth rate advantage from the nutrients is much greater than that of strain *A*. However, the advantage of strain *B* diminishes as the microbial community grows and the effects of lumen nutrients and epithelial nutrients separate into distinct regions. This allows strain *A* to establish itself at the epithelial surface, and for all but the weakest selection by the host, strain *B* is eliminated eventually. In fact, in this example, the host need only provide a 5% growth rate advantage to strain *A* to counter the 100% growth rate advantage and five times higher concentrations that lumen nutrients provide to strain *B*. In summary, we find that a fast growing strain, which would rapidly replace slow growing strains in a well-mixed environment, can be eliminated by moderate counter-selection at the gut epithelium. This process is also effective when strain *A* is initially rare (Figure S4). Host selection at the epithelium, therefore, can effectively operate on an initially rare strain or species that is a minor member of a diverse community.

### Nutrients Are Often Critical to Host Epithelial Selection

We next tested the effects of epithelial selection using antimicrobials that tend to harm strain *B* more than strain *A*. In our model, selection with antimicrobials is slower than with nutrients, because the antimicrobials kill both strains, which reduces the rate at which one strain outgrows the other. Antimicrobials could, in principle, select more quickly than nutrients if they could instantly kill only one of the two strains. In the absence of such extreme selectivity, however, nutrient selection is more powerful. Indeed, for a wide range of conditions, we find that it is critical that the host also supplies nutrients (Figure 4B). These do not need to be selective if selective antimicrobials are secreted. However, nutrients are required because the selective effects of antimicrobials will not permeate up through the community unless there is net positive growth at the epithelial surface. With antimicrobials alone, cell death can easily outweigh the birth of new cells at the epithelial surface because lumen nutrients are at their lowest concentrations. This means that although the host kills more cells of strain *B* than of strain *A* (depending on the specificity of the antimicrobial), if growth is limited by nutrients at the epithelium, no net positive growth of strain *A* will occur either. For this reason, providing nutrients at the epithelial surface greatly widens the range of conditions under which antimicrobials can be used as a selectivity mechanism by allowing sufficient growth in this critical region.

**Figure 5:**
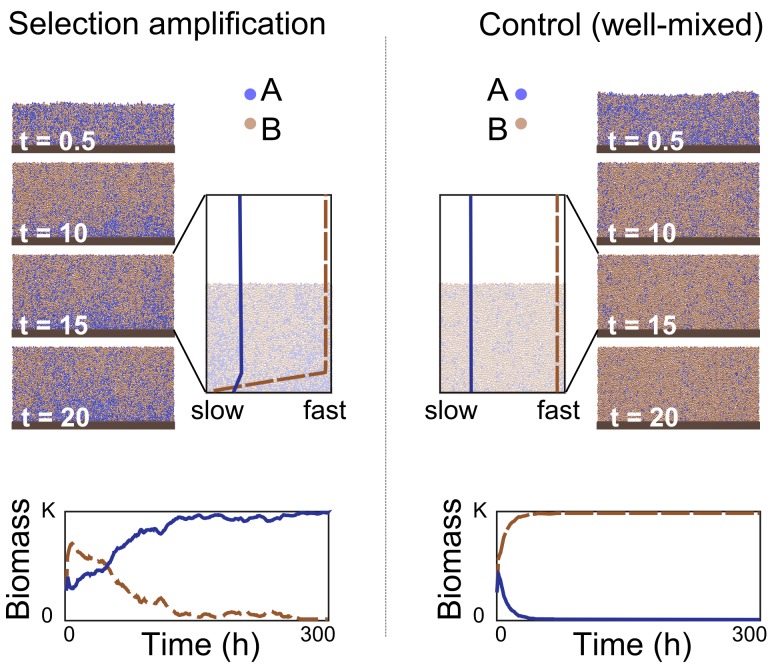
The host need only influence a thin layer of a microbial community to exert control. Selection amplification: To illustrate, we apply constant distributions to all solutes in the simulation (no gradient for lumen nutrients, steep gradient for host secretions) to create a thin layer in which the strain *A* (blue) outgrows strain *B* (brown). The snapshots show the progression of a representative simulation with the expanded snapshot showing the growth rates of the two strains throughout the community. Strain *A* only grows better very close to the surface of the epithelium. Well-mixed: Control simulation with identical total amounts of solutes but without spatial differences in solute concentrations and growth rates of the two strains. In such an environment, strain *A* is out-competed by strain *B*; environmental capacity K.

One challenging case for the host is when lumen nutrient levels are so great as to remove all nutrient gradients in the bacterial community and hence nutrients are available at high concentrations throughout the colony. However, even here, the host can use the epithelium as a selectivity amplifier (Figures 5, S5). Selectivity amplification occurs whenever the host can maintain a thin region next to the epithelium that favours strain *A* over strain *B* and allows for net positive growth. With this, strain *A* will eventually take over the community even though it is counter-selected in the vast majority of the community (Figure 5). As a control, we show in Figure 5 how the same amount of solutes evenly distributed throughout the system would strongly select against *A*, which contrasts with the selectivity amplification seen when solute gradients are present. Finally, our results are robust to fluctuations in lumen nutrient concentrations, which are inevitable in organisms that have discontinuous food intake. As our model predicts, the effects of epithelial secretions are strongest during starvation periods, because lumen nutrient concentrations are highest after feeding [37]. However, implementing a feast–famine cycle that increases the variance in lumen nutrient concentration (but does not affect the mean) suggests that the net effect of these cycles is modest (Figure S6).

## Discussion

The gut is a competitive environment where the potential for high growth rates and population turnover means that slower-growing bacterial strains can be rapidly lost. This presents a problem for hosts. Natural selection of microbial phenotypes based upon intrinsic growth rate will disadvantage any microbes that grow more slowly (Figures 1, 2). Our model predicts that a host can compensate for this effect using epithelial secretions that promote relatively slow-growing strains. Importantly, these effects do not require a highly specific selection mechanism akin to the full force of adaptive immunity. In our model, moderate selectivities that allow poorly growing strains to grow 5% to 10% faster at the epithelial surface are sufficient to reverse their fate.

Epithelial selection may occur either through growth-promoting secretions or toxic growth inhibitors, but we find that growth promotion is often critical because selectivity amplification requires net growth of the microbial community near the epithelial surface. In this context, it is interesting that host epithelial secretions include growth promoters, particularly mucosal glycans [57],[58], in addition to the growth inhibitors of the immune system.

Positive growth at the epithelium surface is important because it causes a flow of microbial cells towards the lumen that limits the effects of lumen nutrients on the community. Cells nearest the lumen are least likely to persist due to detachment and sloughing deeper in the lumen. In our model, this motion is driven by pushing and shoving of dividing bacterial cells. In the mammalian gut, the flow towards the surface is likely to be further promoted by the constant release of mucin polymers from the epithelial surface [57],[59]. Furthermore, the diffusion of IgA—a key secretion known to influence the microbiota—is inhibited by mucins [34]. Our work suggests that this diffusion limitation will maximize not only the residence time of IgA in the gut but particularly the residence time close to the epithelium, where IgA will have an amplified effect.

Our model requires that a host has mechanisms to differentially affect the net growth rate of different bacterial strains or species. Are such effects always possible, particularly in the face of bacterial coevolution to evade the negative effects of host selection? The greatest challenge for host selection will occur when the strains involved are variants of a single species that differ only in their cooperativity towards the host (as opposed to different species that differ in many ways). However, even here, host selection is possible if the host can select directly on the beneficial phenotype in the bacteria [60],[61]. This appears to occur in the mutualism between bioluminescent *Vibrio fischeri* bacteria and the bobtail squid. It is thought that the squid creates an oxidizing environment in the light organ that selects for cells using the luminescence reaction because this reaction uses up oxygen [4].

We believe comparable mechanisms to those seen in the bobtail squid may exist in the gut. Mammalian cells produce glycoconjugates of a remarkable structural complexity and diversity, which are known to favour, or disfavour, the attachment and growth of different microbial species [62]. These compounds may represent an evolutionarily stable way to select for bacteria, like *Bacteroides thetaiotaomicron*, which are carbohydrate specialists that convert complex carbohydrates for the host: *B. thetaiotaomicron* has over five times the number of glycoside hydrolases as species like *Salmonella enterica* or *Shigella flexneri* [63]. Indeed, human milk contains polysaccharides that cannot be digested by the infant, suggesting that mothers may also be exercising this simple but effective form of selection [64]. But is host secretion of complex carbohydrates vulnerable to exploitation by a variant that receives benefits but does not provide any help to the host? The use of complex carbohydrates as a selective mechanism is likely to greatly constrain the evolutionary options for bacterial species by demanding that bacteria use the glycoside hydrolases that also help the host with digestion. Of course, these species might still attempt to invade the epithelial layer. Our model is not intended to capture direct attacks by pathogens, but the detection of tissue damage is a relatively simple problem for a host as compared to selecting among more or less metabolically useful symbionts. And we know that hosts possess mechanisms to counter direct attacks, such as the inflammation response.

However effective, host selection will not preclude bacterial coevolution in the gut. Indeed, long-term bacterial evolution in the gut may allow mutualists to achieve gains in competitiveness both in the presence and absence of host selection. Consistent with such adaptation, *B. thetaiotaomicron* can induce carbohydrate secretion by the host [37]. Coevolution also brings the potential for arms-races with pathogens that adapt and use host-provided nutrients or evade host-secreted antimicrobials. For example, l-Fucose utilization provides *Campylobacter jejuni* with a competitive advantage [65]. More generally, the possibility of bacterial counter-adaptation to host selection mechanisms leaves interesting questions to be answered. These include the issue of how antimicrobial secretions can remain selective when bacteria are known to rapidly develop resistances to many antimicrobials. In addition, the fitness of a bacterial cell will be influenced by cells that possess different secretion, motility, or adhesion phenotypes [66]. We do not yet understand how the potential for complex social interactions among cells will influence host selection. In sum, hosts may be forced to modify or increase their exact selection criteria, either during the life of the host via adaptive immunity or over evolutionary time. Interestingly, recent work has shown how the use of multiple selective mechanisms can allow a host to stay ahead in evolutionary arms races with parasites [67].

Can multiple strains coexist within the epithelial community? We do not find evidence that coexistence is a stable state in our model in the sense that multiple strains will persist indefinitely. This can be seen in Figure 5, where despite lumen selection being much stronger than epithelial selection, the lumen-favoured strain does not persist. The reason for this is that epithelial selection generates a ratchet-like effect whereby the epithelial-favoured population expands and gradually pushes any other strains up and out of the community. If host selection is weak and/or growth in the community is slow, however, favoured and disfavoured strains may both persist for long periods. Moreover, a number of other processes in the gut will counter any winnowing by host selection and help to maintain bacterial diversity. This includes the existence of multiple niches, both at different positions along the epithelial surface but also within the lumen proper. Community diversity will be further influenced by the influx rates of different species [68] and diet [26],[27].

Host epithelial selection is not the only process that influences the microbial species composition of the gut. Nevertheless, our model predicts that the control of epithelium-associated microbial communities is much easier for a host than expected from unstructured environments. Selection of particular microbial species and strains at this position is likely to pay dividends both metabolically but also in terms of the competitive exclusion of undesirable species. Furthermore, epithelium-associated communities are relatively unlikely to be washed out and may represent a stable source community for the rest of the gut. We conclude that host influence on the composition of microbiota is both likely and likely to be powerful.

## Materials and Methods

The study centres upon an individual-based simulation framework that captures bacterial growth and the concentration gradients of solutes, such as nutrients, that originate from bacterial activity while they are growing near to an epithelial host layer. While the model can capture a wide range of conditions, our analysis focuses upon a relatively nutrient-rich environment where cells grow rapidly (Table S1) and slough off at a fixed height above the epithelial surface, which is intended to reflect microbial growth in an animal intestine [32],[52]. In the mammalian gut, these cells will typically grow in the loose upper mucus layer of the epithelial surface, which continually detaches and sloughs off into the lumen [32],[36],[57]. We do not explicitly model the effects of these mucin polymers but implicitly include the protection from sloughing they provide for adherent bacteria in the loose layer. Note that we are only explicitly modelling the bacteria at the surface of the epithelium and not those in the lumen. Of course, selection at the epithelial surface will influence the lumen to some degree (discussion), but we do not explicitly model this process.

The model is an extension of an established framework that has been developed and tested over the last 15 years to understand and predict the behaviour of bacterial communities growing on inert surfaces [54],[69]–[73]. While originally developed for problems in bioengineering, it has most recently been applied to understand the evolution and ecology of microbial groups [55],[66],[73]–[75]. Subsequent empirical validation of these models has demonstrated the ability of the framework to both describe bacterial communities and identify new biological mechanisms [76],[77]. The model assumptions, justifications, and implementation are extensively discussed elsewhere [69],[70],[72],[78]. In brief, bacterial cells are modelled as solid spheres that metabolise nutrients in a continuous concentration field. At each iteration, the concentration field is updated solving the two- or three-dimensional reaction-diffusion equations using multigrid solvers. This takes into account local sinks, such as a bacterium utilising the solutes around it as a nutrient source or local sources, such as secretions from a cell. Cells increase in diameter and eventually divide pushing aside neighbouring cells.

The model focuses upon the resident bacterial communities that grow in the loose upper mucus layer at the interface of the lumen and epithelium, which are most likely to be affected by host selection [79]. We inoculate our simulations with a total of 250 cells in varying frequencies. This is a simplification as initial assembly of the microbiota has been shown to be more complex and may depend on interbacterial cross-talk as well as other yet unknown factors [80]. Bacteria reside above a layer of host cells that secrete solutes at varying rates. We assume that this epithelial layer and the dense mucus layer immediately above it is impenetrable to the bacterial cells [32],[81]. This is supported by data on the healthy gut with a few notable exceptions, such as segmented filamentous bacteria in mice that live in the dense mucus layer [41]. Accordingly, we do not consider host responses to invasion of a pathogen or breach of the mucus layer, such as inflammation (but see Discussion). The bacteria grow and divide utilising nutrients diffusing in from the lumen or the epithelium. At a certain height above the epithelium, cells are sloughed and excluded from the simulation. Bacteria utilise nutrients (*N*) and convert them into biomass at the rate *μ* following Monod-kinetics:
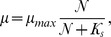
where *K_s_* is the Monod constant. Competing strains in our simulations differ in their maximum growth rates, *μ_max_*. Bacteria may switch between different substrates, ensuring that the maximum growth rate cannot be exceeded, where switching is based upon a recent analysis of optimal foraging in microbes [82]. Death of cells through antimicrobials is modelled using a similar equation as for growth:
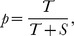
where *p* is the probability of death for a cell, *T* is the local concentration of the antimicrobial, and *S* the concentration at which cell death within 1 h occurs with a probability of 50%. Different strains may have different susceptibilities to the antimicrobial and hence different probabilities for cell death at a given concentration. Most of our understanding of host-secreted antimicrobials stems from secretions of the epithelium in the small intestine, whereas secretions in the larger intestine are less well understood [41],[48]. 

## Supporting Information

Figure S1 Diagram of the simulation setup. Cells live and divide on an impenetrable host epithelium. Growth-promoting nutrients can diffuse into the bacterial colony from the lumen (top) and/or the epithelium (bottom) where they are utilized by the cells. In some simulations the epithelium also releases antimicrobials that kill cells. The direction of fluxes is indicated by arrows (nutrients, green; antimicrobials, red). Periodic boundaries at the sides simulate continuous space. Cells moving beyond the maximum thickness are removed simulating sloughing (dashed line, the location is a parameter that we vary).
(TIFF)

Figure S2 Final frequencies of faster growing strain *B* in a simplified ordinary differential equation model. A minimum final frequency of the faster growing strain is found for intermediate microbial community thickness; the exact location depends on the growth functions of the two strains (see Text S1).
(TIFF)

Figure S3 The effect of non-selective nutrients from one direction on selectivity from the other direction. We show the biomass development over time beginning with cells at low densities (125 cells each). (A) Host-secreted nutrients provide a growth rate advantage and neutral nutrients diffuse into the biofilm from the lumen. (B) Lumen nutrients provide a growth rate advantage and the host secretes neutral nutrients. Points of sloughing are 20, 40, and 80 µm. For 80 µm, lumen selection is strongly impeded by the presence of neutral host nutrients, whereas host selection is unaffected by additional neutral lumen nutrients (initially the favoured species outgrows the other but upon reaching the capacity will be sloughed off more frequently, leading to a decrease in frequency compared with the maximum at ∼10 h).
(TIFF)

Figure S4 Selection amplification of an initially rare strain by the host epithelium. Weak epithelial selection dominates strong lumen selection. Strain *B* has a 100% growth rate advantage on nutrients from the lumen, and lumen nutrients are five times the concentration as epithelial nutrients. Host nutrients provide growth rate advantages to an initially rare strain *A* (initial frequency 0.1, grey plane) ranging from 1% to 100%.
(TIFF)

Figure S5 Selection with antimicrobials. Selection via host antimicrobial secretion in the absence of host nutrient secretion is possible only when lumen nutrients are available throughout the bacterial colony. The host can select for slow growing strain *A* despite antimicrobials being available at relevant concentrations only in a fraction of the overall bacterial colony (near the epithelium) when selectivities of antimicrobials in favour of the strain *A* (S_B_<S_A_) are sufficiently high. In the majority of the bacterial colony, cells of strain *B* have a net growth rate advantage due to the high concentration of lumen nutrients, which favour *B*. Capacity K, maximum biomass.
(TIFF)

Figure S6 Fluctuations in lumen nutrient concentrations do not affect predictions. Each figure shows the biomass development of the two strains in 30 independent simulations (brown, strain *B*; blue, strain *A*) with discontinuously available nutrients in the lumen. Feast-famine periods last 8 h each. For comparison, thick black lines show the mean biomass from 30 simulations under identical selection strengths but with continuously available nutrients for stain *B* (dashed) and strain *A* (solid). Host nutrients provide varying growth rate advantages to strain *A* as indicated in the figure. The mean nutrient concentration in the lumen is five times higher than nutrients from the host, and strain *B* has a 100% growth rate advantage over *A* on these nutrients. Mean nutrient concentrations in the continuous and discontinuous case are identical. Capacity K, maximum biomass.
(TIFF)

Table S1 Simulation parameters. L, length; M mass; T, time.
(DOC)

Text S1 Simplified ordinary differential equation model. The model shows the occurrence of a minimum influence of the lumen on the outcome of competition in the individual-based simulations for intermediate bacterial colony thicknesses.
(PDF)

## Abbreviations

IgA: Immunoglobulin A; ODE, ordinary differential equation
