# Supplementary figures and images for: The Evolution of Mutualism in Gut Microbiota Via Host Epithelial Selection
Source: PLoS Biol. 2012 Nov 20;10(11):e1001424. doi: 10.1371/journal.pbio.1001424 (PMC3502499; doi:10.1371/journal.pbio.1001424)

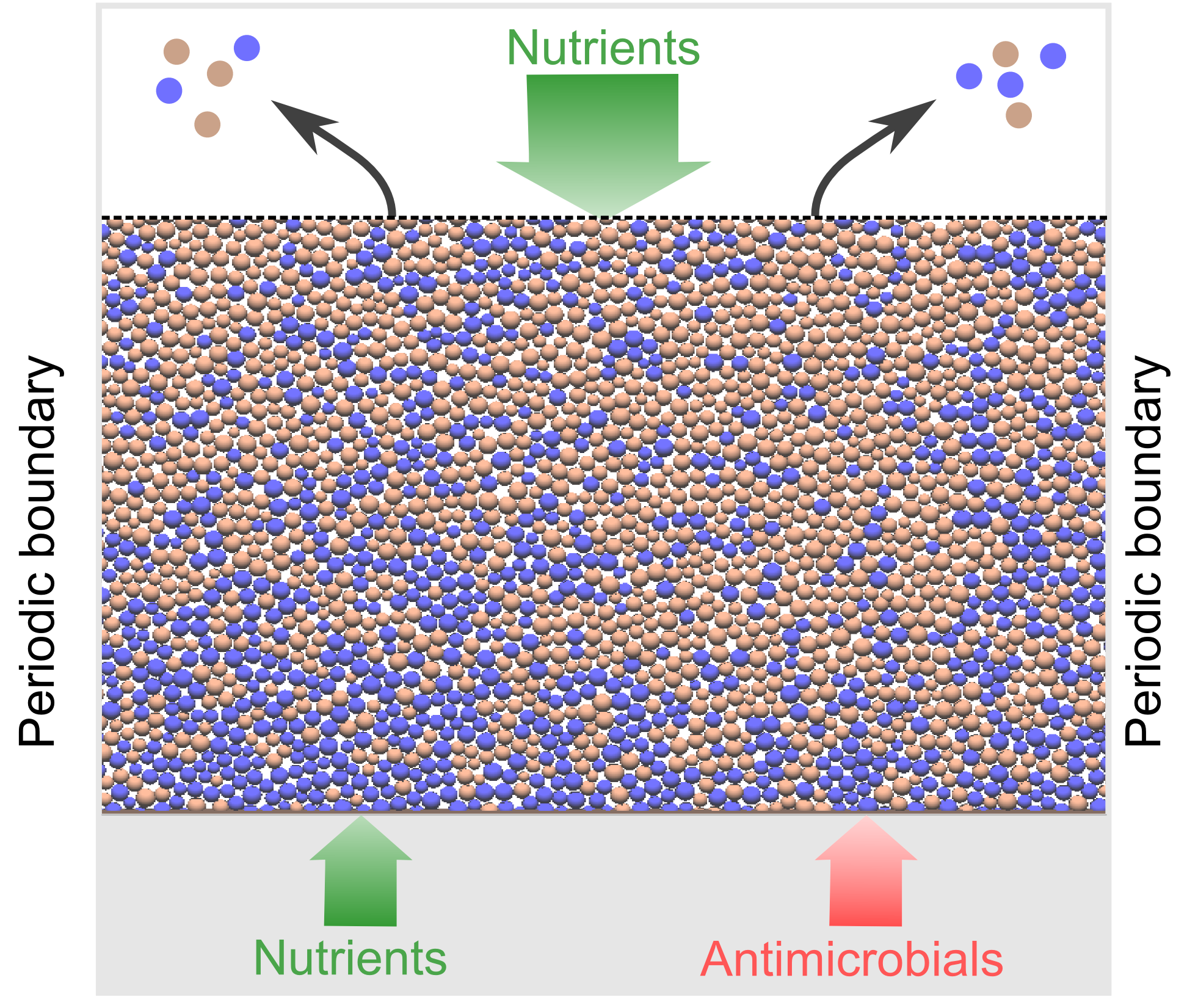

Supplement: Figure S1 — Diagram of the simulation setup. Cells live and divide on an impenetrable host epithelium. Growth-promoting nutrients can diffuse into the bacterial colony from the lumen (top) and/or the epithelium (bottom) where they are utilized by the cells. In some simulations the epithelium also releases antimicrobials that kill cells. The direction of fluxes is indicated by arrows (nutrients, green; antimicrobials, red). Periodic boundaries at the sides simulate continuous space. Cells moving beyond the maximum thickness are removed simulating sloughing (dashed line, the location is a parameter that we vary). (TIFF) [file pbio.1001424.s001.tif]

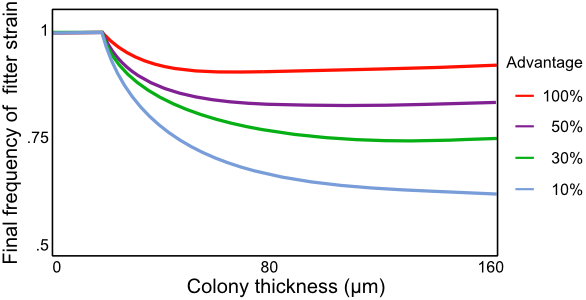

Supplement: Figure S2 — Final frequencies of faster growing strain B in a simplified ordinary differential equation model. A minimum final frequency of the faster growing strain is found for intermediate microbial community thickness; the exact location depends on the growth functions of the two strains (see Text S1). (TIFF) [file pbio.1001424.s002.tif]

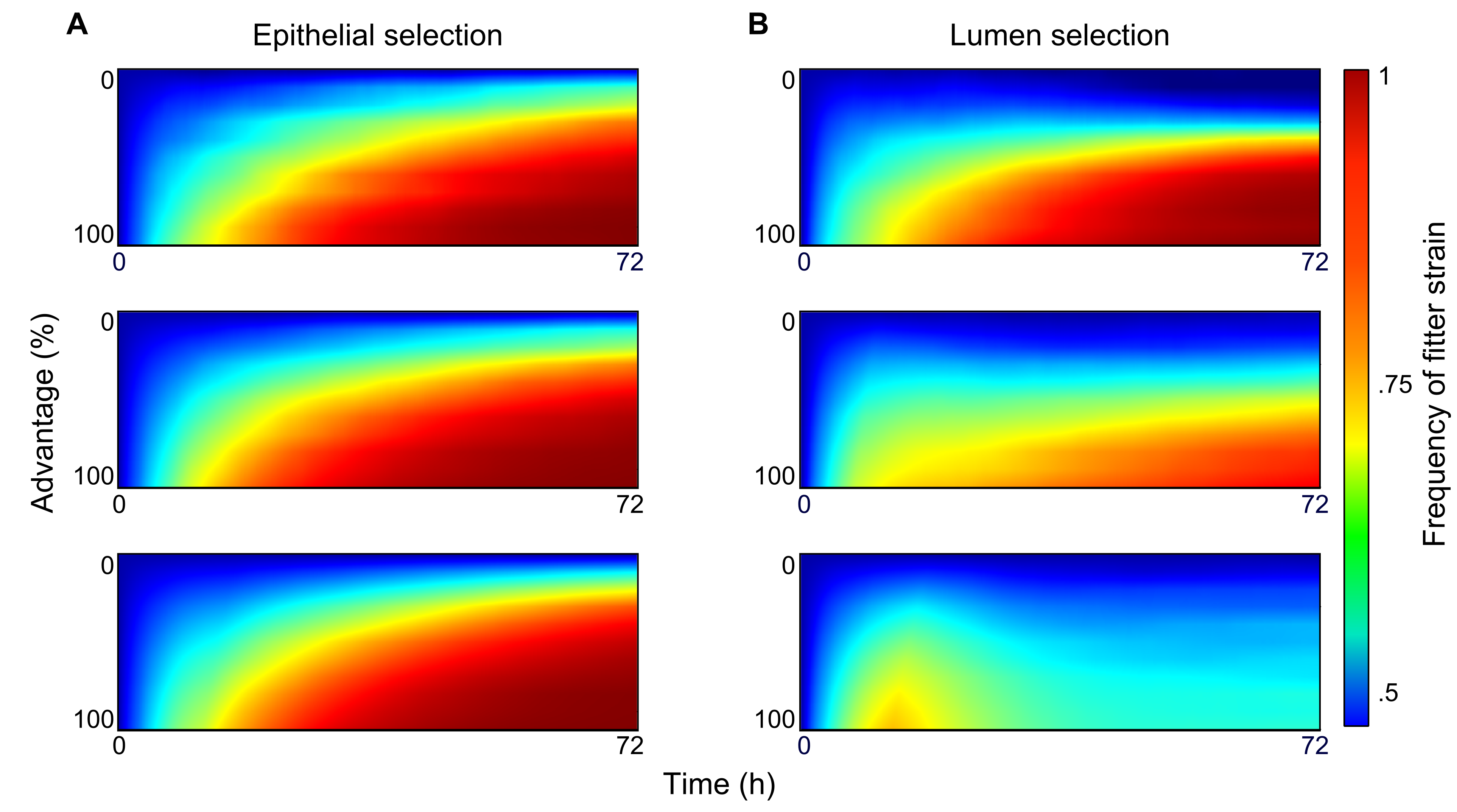

Supplement: Figure S3 — The effect of non-selective nutrients from one direction on selectivity from the other direction. We show the biomass development over time beginning with cells at low densities (125 cells each). (A) Host-secreted nutrients provide a growth rate advantage and neutral nutrients diffuse into the biofilm from the lumen. (B) Lumen nutrients provide a growth rate advantage and the host secretes neutral nutrients. Points of sloughing are 20, 40, and 80 µm. For 80 µm, lumen selection is strongly impeded by the presence of neutral host nutrients, whereas host selection is unaffected by additional neutral lumen nutrients (initially the favoured species outgrows the other but upon reaching the capacity will be sloughed off more frequently, leading to a decrease in frequency compared with the maximum at ∼10 h). (TIFF) [file pbio.1001424.s003.tif]

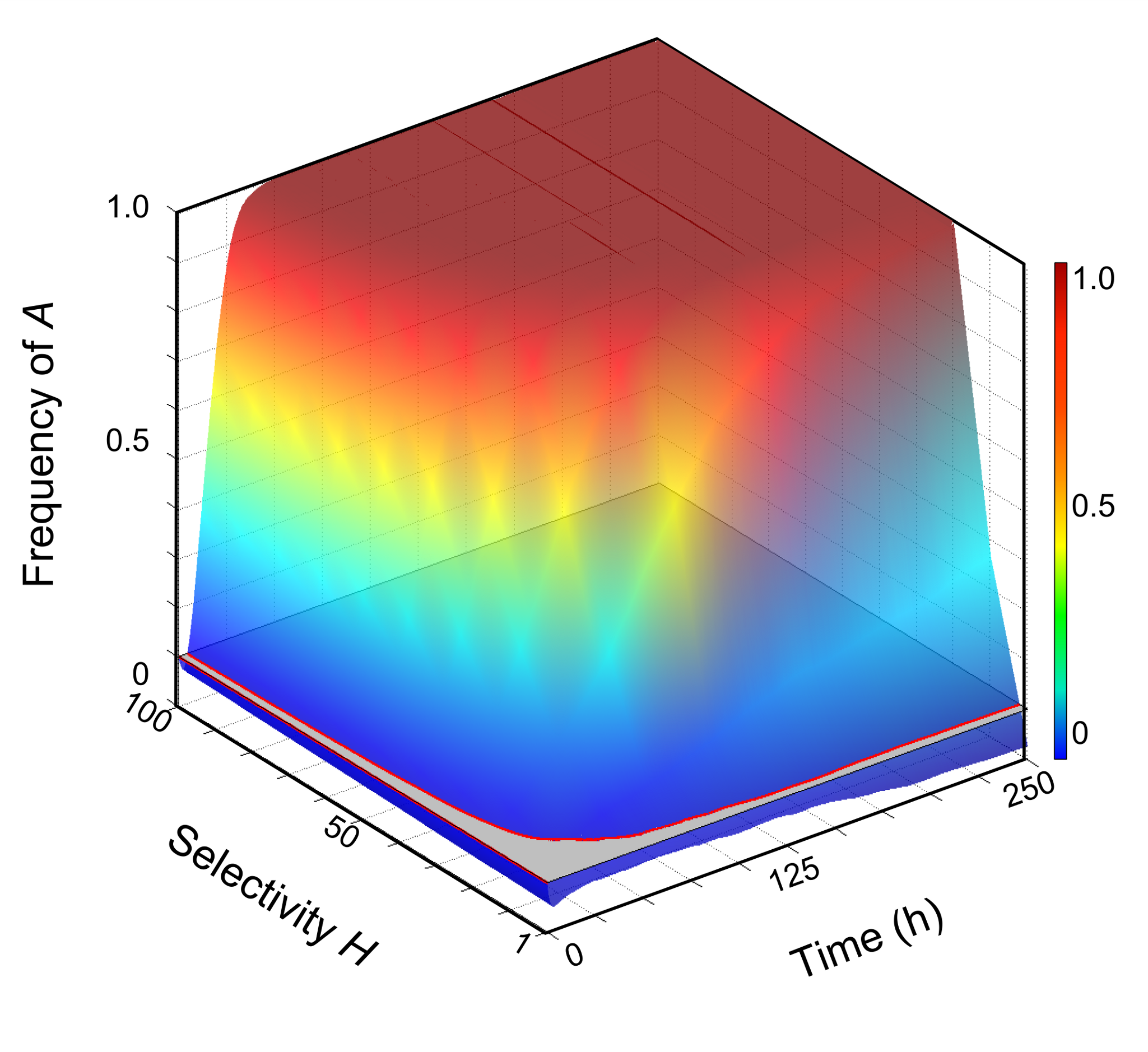

Supplement: Figure S4 — Selection amplification of an initially rare strain by the host epithelium. Weak epithelial selection dominates strong lumen selection. Strain B has a 100% growth rate advantage on nutrients from the lumen, and lumen nutrients are five times the concentration as epithelial nutrients. Host nutrients provide growth rate advantages to an initially rare strain A (initial frequency 0.1, grey plane) ranging from 1% to 100%. (TIFF) [file pbio.1001424.s004.tif]

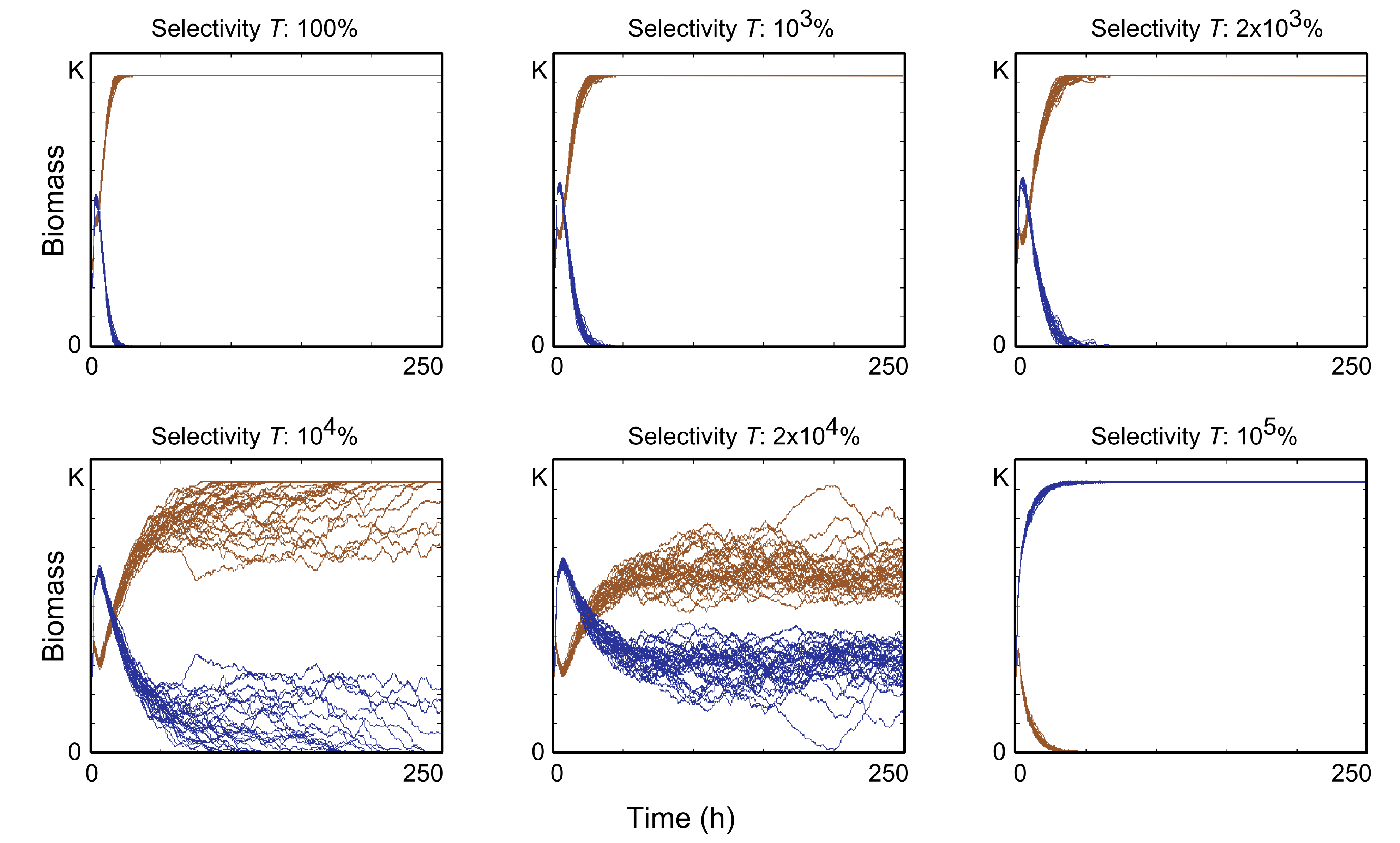

Supplement: Figure S5 — Selection with antimicrobials. Selection via host antimicrobial secretion in the absence of host nutrient secretion is possible only when lumen nutrients are available throughout the bacterial colony. The host can select for slow growing strain A despite antimicrobials being available at relevant concentrations only in a fraction of the overall bacterial colony (near the epithelium) when selectivities of antimicrobials in favour of the strain A (SB<SA) are sufficiently high. In the majority of the bacterial colony, cells of strain B have a net growth rate advantage due to the high concentration of lumen nutrients, which favour B. Capacity K, maximum biomass. (TIFF) [file pbio.1001424.s005.tif]

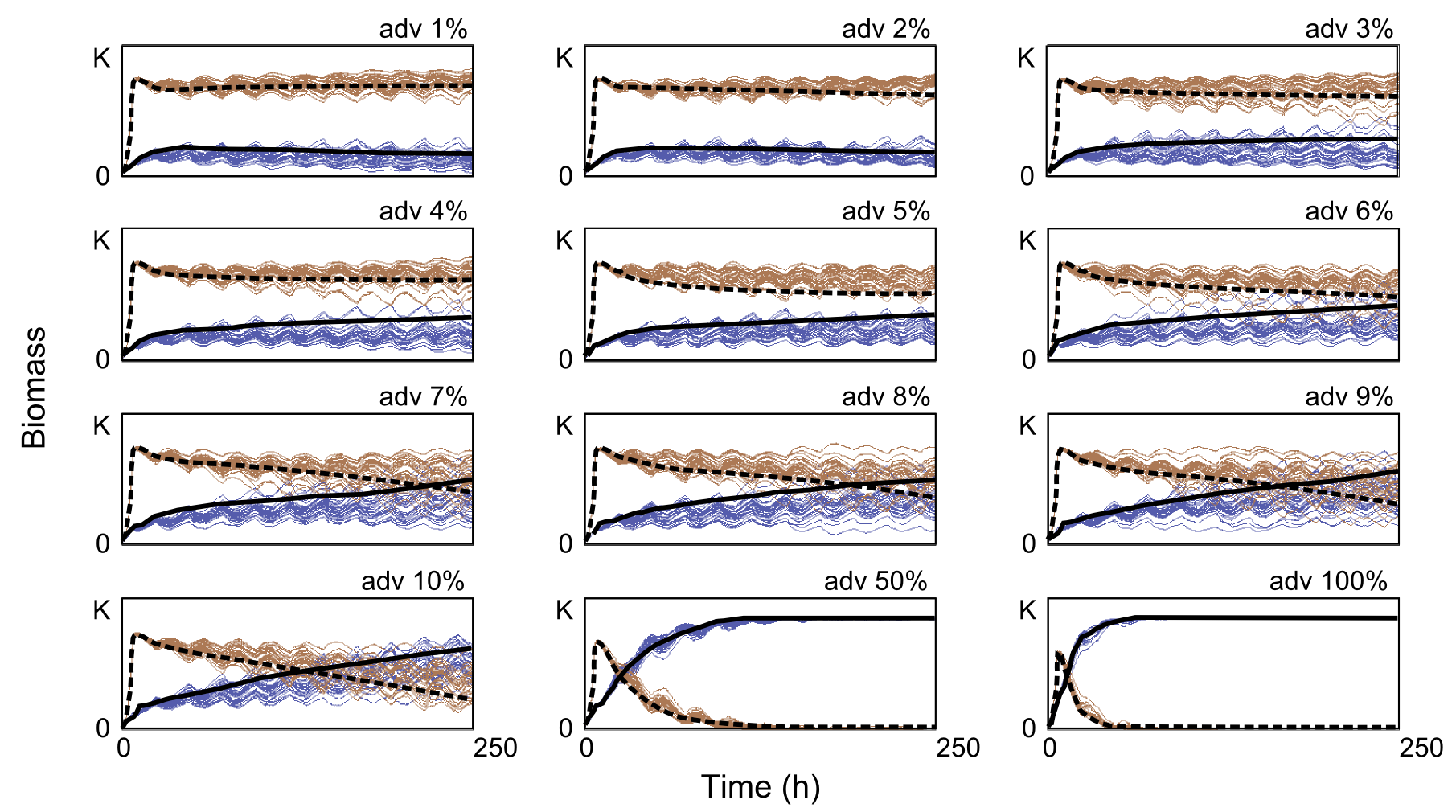

Supplement: Figure S6 — Fluctuations in lumen nutrient concentrations do not affect predictions. Each figure shows the biomass development of the two strains in 30 independent simulations (brown, strain B; blue, strain A) with discontinuously available nutrients in the lumen. Feast-famine periods last 8 h each. For comparison, thick black lines show the mean biomass from 30 simulations under identical selection strengths but with continuously available nutrients for stain B (dashed) and strain A (solid). Host nutrients provide varying growth rate advantages to strain A as indicated in the figure. The mean nutrient concentration in the lumen is five times higher than nutrients from the host, and strain B has a 100% growth rate advantage over A on these nutrients. Mean nutrient concentrations in the continuous and discontinuous case are identical. Capacity K, maximum biomass. (TIFF) [file pbio.1001424.s006.tif]
