# Supplementary material for: The Evolution of Mutualism in Gut Microbiota Via Host Epithelial Selection
Source: PLoS Biol. 2012 Nov 20;10(11):e1001424. doi: 10.1371/journal.pbio.1001424 (PMC3502499; doi:10.1371/journal.pbio.1001424)
Supplement: Table S1 — Simulation parameters. L, length; M mass; T, time. (DOC) [file pbio.1001424.s007.doc]

**Table S1)** **Simulation parameters.** Length (L), mass (M), time (T).

| Symbol | Description | Dimension | Value | Units | References |
| --- | --- | --- | --- | --- | --- |
|  | Max. colony thickness | L | 10-160 | μm | [32] |
|  | Max. cell radius before division | L | 1 | μm | [74] |
|  | Width of simulated colony | L | 250 | μm |  |
|  | Initial cell number (*A*+*B*) |  | 250 |  |  |
|  | Boundary layer thickness | L | 25 | μm | [74] |
| *μ_i,N/L_* | maximum growth rates of strain *i* on nutrient *N* or *L* | T^-1^ | 0.5 ≤ *μ* ≤ 1 | h^-1^ | [52,74] |
| *K_N,L_* | Half saturation constant for growth on nutrient *N* and *L* | ML^-3^ | 3.5*10^-5^ | gl^-1^ | [55,74] |
| *D* | Diffusion coefficient of solutes | L^2^T^-1^ | 4*10^4^ | μm^2^h^-1^ | [55,74] |
| *L* | Bulk lumen nutrient concentration | ML^-3^ | 0.8, 4 or  non-limiting (const. at 4) | gl^-1^ | Based on *high nutrient concentration* in [74] |
| *H* | Maximum host nutrient concentration near the epithelium. The secretion rate of nutrients from host epithelial cells is based on total fluxes from the lumen such that energy values can be compared, i.e. five times more from the lumen in Figure 4. | ML^-3^ | ≤ 0.8 | gl^-1^ |  |
| *S* | Susceptibility defined as concentrations at which cell death within one hour occurs with a probability of 50%. Values based on maximum antimicrobial concentration (*T*) such that cell growth is possible. | ML^-3^ | 0.065-0.26 | gl^-1^ |  |
| *T* | Maximum concentration of antimicrobial near the epithelium | ML^-3^ | 0.005 | gl^-1^ |  |
| *ρ* | Density of bacterial biomass | ML^-3^ | 150 | gl^-1^ | [74] |
| *Y* | Yield of biomass per substrate |  | 0.5 |  | [74] |
