# Supplementary material for: The Evolution of Mutualism in Gut Microbiota Via Host Epithelial Selection
Source: PLoS Biol. 2012 Nov 20;10(11):e1001424. doi: 10.1371/journal.pbio.1001424 (PMC3502499; doi:10.1371/journal.pbio.1001424)
Supplement: Text S1 — Simplified ordinary differential equation model. The model shows the occurrence of a minimum influence of the lumen on the outcome of competition in the individual-based simulations for intermediate bacterial colony thicknesses. (PDF) [file pbio.1001424.s008.pdf]

Text S1

We simplify the individual based simulations where nutrients exclusively come from the lumen by considering two non-spatial, coupled ordinary differential equations describing the biomass development of strain *A* and *B* over time. Starting from an initial monolayer of cells, the bacterial colony in the individual based model increases in thickness due to cell division, eventually excluding cells below from access to nutrients. The region of active growth where nutrients are available at sufficient concentrations moves upwards with the growing colony towards the source of nutrients in the lumen. When the maximum height (which we vary) where cells are sloughed off is reached, a region of actively dividing cells exists, which we will call final active layer. Here, cells are removed constantly through sloughing and replenished by bacterial growth. We can describe the competition between strain *A* and *B* in the active layer with

$$\frac{dA}{dt} = \mu_{maxA}\left(1 - \frac{A+B}{\alpha}\right)A - \varsigma A \quad (1)$$

$$\frac{dB}{dt} = \mu_{maxB}\left(1 - \frac{A+B}{\alpha}\right)B - \varsigma B \quad (2),$$

where $\mu_{maxB} > \mu_{maxA} > \varsigma > 0$. Under these conditions, the frequency of *B* tends towards 1 as constant removal of a fraction of cells due to sloughing (ς) and faster replenishing of *B* will lead to successful competition for the limited capacity (α) of the final active layer. Before the colony has reached it's final thickness, regions below the final active layer are subject to competition between the two strains simplified as

$$\frac{dA}{dt} = \mu_{maxA}\left(1 - \frac{A+B}{K}\right)A \quad (3)$$

$$\frac{dB}{dt} = \mu_{maxB}\left(1 - \frac{A+B}{K}\right)B \quad (4),$$

with capacity of the system *K*, which is solved until $t^* = 72$ days equivalent to the individual

based simulations. The final frequency $R(t^*)$ of B (shown in Figure S2) is calculated taking into account the frequency of B below the final active layer at t^* and the fraction of the total colony that lies within the final active layer where the frequency of B will be 1 (eqs. 1 and 2). Hence,

$$R(t^*) = \frac{B(t^*)}{A(t^*) + B(t^*)} \frac{K - \alpha}{K} + 1 \frac{\alpha}{K} \quad (5),$$

where K is the overall capacity of the colony which increases with the thickness and α is the capacity of the final active layer which is constant and $K \geq \alpha$. Hence the contribution to the final frequency of B from α in the last term in equation 5 is larger for smaller K, i.e. in thinner colonies. The values for K and α are based on the values of total biomass found in the individual based simulations for different points of sloughing. For values of K smaller than α (thin colonies), the final ratio is 1, as the entire bacterial colony is subject to constant selection because nutrients are available everywhere, i.e. the entire bacterial colony resides within the final active layer.

These considerations allow us to distinguish three compartments of a steady state bacterial community: the final active layer (*AL*) which will exist for any sloughing height, and two other compartments below *AL*, which can arise depending on where sloughing occurs. In the early stages, strains *A* and *B* exist in a 50:50 ratio. Then, in these simulations, because $\mu_{maxB} > \mu_{maxA}$, strain *B* begins to outgrow strain *A* and will eventually take over the surface of the growing colony entirely (Sketch, B). Hence, all cells with access to nutrients will be of strain *B*. Immediately below the final active layer, there thus may then be a region which is comprised only of cells of strain *B* (non-dividing as no nutrients can reach this region). We call this compartment *Q*. Below *Q* at the base of the colony, another stagnant region exist which unlike *Q* is comprised of both strains (compartment *W*).

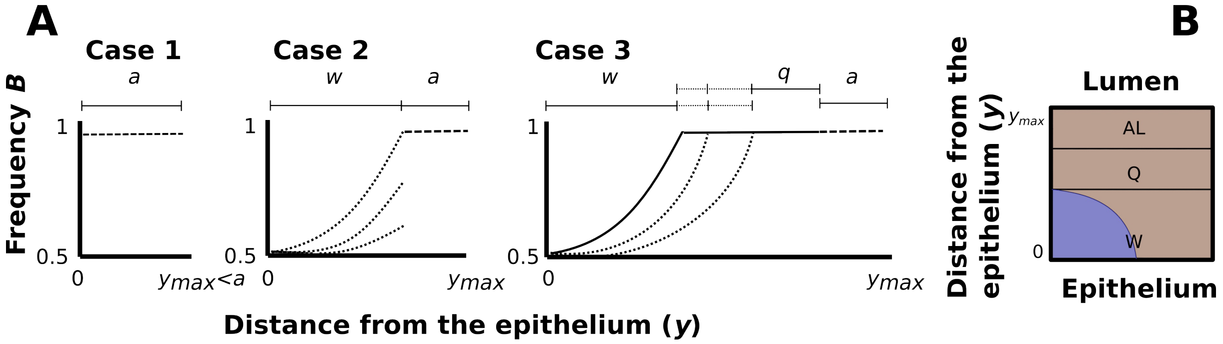

Sketch: A) Graphs show the frequency of strain *B* as we move away from the epithelium. Lower case *a*, *q*, *w* indicate the location of the compartments *AL*, *Q*, *W*. Case 1) In thin bacterial colonies, nutrients are available everywhere and the entire colony [0 *y*] lies within the final active layer (*a*). Case 2) The final active layer is located at the top of the colony below which a compartment where strain *B* and *A* coexist arises (*w*). Case 3) In thick colonies, a third compartment arises (*q*) which harbours stagnant cells of strain *B* only. Different curves result from different growth functions and growth rate advantages for *B*. B) Cartoon demonstrating the biomass distribution in a two dimensional colony for case 3 (strain *A* blue, *B* brown). Compartments AL, Q and W arise: *AL*, a stagnant region only containing *B* cells: *Q*, and at the base a stagnant region containing a mixture of *A* and *B* cells: *W*.

How does altering the colony thickness (y) affect final total ratios? We call the distance from the epithelium of the three compartments a, q and w. For sloughing points very close to the epithelium, nutrients will always penetrate the entire system ($y \leq a$) and hence the entire colony is subject to continuous selection and will thus tend to be composed entirely of the faster growing strain B (Case 1). Colony thicknesses for which case 1 holds will result in a final frequency of B of 1. When compartmentalisation due to nutrient depletion at the base of the colony arises ($y > a$), we declare $w = y - a$. Competition between the two strains leads to an increase in the frequency of the faster growing strain B. As we move away from the epithelium where the initial frequency is 0.5, we can describe the increase in frequency of B as a function $g(y)$ of the distance (y) from the epithelium between 0 and w (from the epithelium to the active layer). Case 2 arises when $0 < w <= y^*$, where $y^* := g(y) = 1$. Hence y^* is the distance from the epithelium at which B has taken over entirely. For case 2, the

final ratio is given by:

$$R_{C2} = 1 - \frac{0.5w - \int_0^w g(y)dy}{w + a} \qquad (6).$$

Case 3 finally arises for $y - a > y^* = w$. In case 3, we find all three compartments described above and the final frequency of *B* is given by

$$R_{C3} = 1 - \frac{0.5y^* - \int_0^{y^*} g(y)dy}{y^* + q + a} \qquad (7)$$

The colony thickness for which the frequency of *B* is minimal will be intermediate as it has to be larger than *a* and (7) tends to 1 again for very large y_{max} (and thus very large *q*). The exact location of the minimum will depend on the function *g(y)*.
